# Pharmacological Evaluation of* Chrozophora tinctoria* as Wound Healing Potential in Diabetic Rat's Model

**DOI:** 10.1155/2016/7475124

**Published:** 2016-12-20

**Authors:** Harikesh Maurya, Monika Semwal, Susheel Kumar Dubey

**Affiliations:** Siddhartha Institute of Pharmacy, Sahastradhara Road, Dobachi, Dehradun 248001, India

## Abstract

*Objective*. The study was designed to evaluate pharmacological potential of hydroalcoholic leaves extract of* Chrozophora tinctoria* intended for wound healing in diabetic rats' model.* Methods*. The method used to evaluate the pharmacological potential of hydroalcoholic leave extract was physical incision rat model. In this model, cutting of the skin and/or other tissues with a sharp blade has been made and the rapid disruption of tissue integrity with minimal collateral damage was observed shortly. Animals used in the study were divided into four groups that consist of six animals in each group. Group I serves as normal control, Group II serves as disease control, Group III was used as standard treatment (Povidone iodine 50 mg/kg b.w.), and Group IV was used for test drug (*C. tinctoria* 50 mg/kg b.w.).* Result*. The hydroalcoholic leave extract of* Chrozophora tinctoria* has been significantly observed to heal the wound (98%) in diabetic rats within 21 days, while standard drug (Povidone iodine) healed the wound about 95% in the same condition. The oral dose (50 mg/kg b.w.) of* Chrozophora tinctoria* was also found to improve the elevated blood glucose level in comparison to disease control group, which increased after the oral administration of Streptozotocin.* Conclusion.* The* Chrozophora tinctoria* has significant wound healing potential in the animal having physically damaged tissue in diabetic condition.

## 1. Introduction

Wound healing is a complex progression, where the skin or other body tissues maintain themselves behind injury. In physical wound, the epidermis and dermis layers of the skin show a defensive obstruction at the side of the exterior atmosphere [1, 2]. When the obstruction is not working, an orchestrated cascade of biochemical measures is rapidly placed into motion to renovate the injury [3]. This procedure is divided into passionate direction of expected phases, that is, blood clotting (hemostasis), inflammation, the enlargement of new tissue (proliferation), and the remodeling of tissue (maturation) [4, 5].

*Chrozophora tinctoria* is an annual plant belonging to family Euphorbiaceae and exclusively described as a species in 1824 and comprises monoecious herbs or undershrubs [6]. The species of this plant is well known across Europe, Africa, and Asia.* Chrozophora tinctoria* is a yearly level to the ground herb widespread of dry waste spaces on sandy clay and the flowering condition observed in the months of April to June [7]. It can grow up in semishade (light woodland) or having no shade with required wet soil [8]. It is identified as dyer's croton, giradol, or turnsole and is inhabitant to Africa, tropical Asia, and Europe [9]. In Iran, the plant is used to care for warts, emetic, cathartic, and fever whereas root ashes are given to children for cough. The seeds are purgative or cathartic, even though its bark is used for tanning and coloring [10].

Naturally the various useful chemical constituents present in the leaves of herbal plants are playing an important role in the field of pharmacological behavior on living system [11, 12]. The* C. tinctoria* plant is monoecious, and indumentums consist of extremely intense, sessile, and peduncle stellate or lepidote hairs, next to simple hairs. Stipules narrowly triangular, scars are exceedingly indistinct [13]. Almost 150 kinds of plant are used in the manufacture of ordinary dyes. Nearby immense amplification in the amount of autecological studies of plants worn during normal dye manufacture is called the turnsole plant. It is worn to care for warts despite the fact that leaves are used during chest burning into Kadhi areas of Khushab [14]. The study was designed to find out the therapeutic potential of* Chrozophora tinctoria* leaves extract for the management of wound healing in diabetic conditions.

Internationally published report suggested that the* Chrozophora tinctoria* yielded five flavonoid glycosides, (1) quercetin 3-O-rutinoside (rutin), (2) acacetin 7-O-rutinoside, (3) apigenin 7-O-b-D-[(6-p-coumaroyl)]-glucopyranoside, (4) apigenin 7-O-b-D-glucopyranoside, and (5) apigenin 7-O-b-D-[6-(3,4-dihydroxybenzoyl)]-glucopyranoside (chrozophorin) with the last one being a new natural product [15].

## 2. Materials and Methods

### 2.1. Collection and Authentication of Plant

The herbal plant* C. tinctoria* is collected in the months of May-June from the district of Ambedkar Nagar, Uttar Pradesh (India) [16], for the examination of wound healing potential. Fresh and shade dried leaves have been collected consequently and authenticated by Botanical Survey of India, Northern regional centre 192, Kaulagarh road, Dehradun 248195, with plant Acc. number 114546.

### 2.2. Preparation of Extract

The fresh shade dried leaves of* C. tinctoria* (1000 g) were collected separately and powdered for the extraction. The hydroalcoholic agents successively with 80% methanol and 20% water were used for extraction of collected parts by Hot Maceration process (Soxhlet apparatus) [17]. After extraction it was dried to make powder form and stored in well tight closed container. The obtained powdered drug was subjected for the study of pharmacological activity on rats [18].

### 2.3. Preparation of Dose

The selected dose of the test drug (*C. tinctoria* extract 50 mg/kg b.w.) 5% solution and the standard drug (Povidone iodine ointment 50 mg/kg b.w.) 5% solution was carefully prepared. The oral doses of the test and the standard drug were prepared with the dose of 50 mg/kg b.w. for the measurement of blood glucose level. The selected animal model was grouped and administered with prepared drug accordingly for the wound healing activity [19].

### 2.4. Preliminary Phytochemical Analysis

A preliminary phytochemical screening was carried out for the* C. tinctoria* leaves extract employing the standard procedure, and it reveals the presence of alkaloids, mucilage, anthraquinone, saponins, tannins, flavonoids, steroids, terpenoids, glycosides, and reducing sugar [20].

### 2.5. Experimental Animal

Albino Wistar rats (150–180 g) of either sex were used for the study. These animals were maintained under the controlled conditions of temperature (25 ± 2°C), humidity (55 ± 5%), and 12 : 12 h light dark cycle. All the animals were acclimatized for at least two weeks before the study. The animals randomized into test and control groups were housed individually in sanitized polypropylene cages containing sterile paddy husk as bedding. They were freely assessed to standard pellets as basal diet and water add libitum. All animals were habituated to laboratory conditions for 48 h prior to experimental protocol to minimize any nonspecific stress, which was followed by CPCSEA guidelines. The experimental protocol was approved by the Institutional Animal Ethical Committee (Approval number SIP/IAEC/PCOL/01/2016) and CPCSEA Registration number 1435/PO/Re/S/11/CPCSEA.

### 2.6. Experimental Design

All animals were divided into four groups consisting of six animals in each group: Group I, normal control (given standard diet and water for 3 weeks); Group II, disease control (given Streptozotocin 45 mg/kg b.w. single dose and wound by physically damaging the tissue); Group III, Streptozotocin (45 mg/kg b.w. single dose) + standard drug (Povidone iodine 50 mg/kg b.w. applied every day for 3 weeks); and Group IV, Streptozotocin (45 mg/kg b.w. single dose) + test drug (CT leaves extract 50 mg/kg b.w. applied every day for 3 weeks) followed by standard diet and water for all groups. Oral doses of 50 mg/kg b.w. of each standard and test drugs were also administered to Groups III and IV rats every day for 3 weeks. These animals were caged separately according to the groups.

### 2.7. Acute Toxicity Study

The staircase method was adopted for the determination of acute toxicity. Albino Wistar rats of either sex weighing 180–220 g of 60 days of age were used to determine the safer dose according to OECD guideline.

### 2.8. Model Used for Wound Activity

The standard excision model for wound was selected to observe healing activity. All the rats were anaesthetized with ether and the exposed part on back was saved with a sharp blade [21]. A circular piece (500 mm^2^ area) was impressed on the dorsal thoracic region 5 cm away from head. The animals were individually housed in separate cages. The cutting skin/tissue results in rapid disruption of the tissue integrity with minimal collateral damage [22]. The amount of gap in the incision has depended on the amount of subcutaneous fat and the tensional forces on the wound site. Wound contraction was monitored by measuring wound area with 7-day gap till 21st postoperative day [23].

During the experiment no local or systemic antimicrobial agents were used, while the wound was left undressed. The full aseptic measures were also not taken throughout the experiment [24]. In favor of the observation of blood glucose level and wound healing activity, one group was treated with the* C. tinctoria* ointment (5% solution); the second group was treated with Povidone iodine ointment (5% solution) by both oral and dermal routes of administration; tensile strength was determined on 14th day as postwounding [25]. Tensile strength, the force required to open a healing skin wound, was used to measure healing. The instrument for this measurement is called Tensiometer. It consists of a 6 × 12-inch board with one post of 4 inches long fixed on each side of the longer ends [13].

The percentage of wound healing was calculated by the given formula:

(1) %  Wound  healed=Wound  area  on  day1−Wound  area  on  daynWound  area  on  day1×100,

where *n* = number of days.

### 2.9. Biomarkers for Wound Healing

Subsequent biomarkers which have been observed in this study were body weight, body temperature, and animal behavior in favor of physical observation, while the blood glucose level was analyzed for laboratory investigation. Biochemical parameters like granulocytes, protein count, collagen content, tensile strength, and epithelialization period were observed as the main investigation of the experimental result and histopathology of the wound tissue for its confirmation.

### 2.10. Statistical Analysis

All biological parameters monitored out during the experiment of each separated group were collected consequently. All the collected data were expressed as mean ± SEM, by using Graph Pad Prism-5. *P* < 0.05 has been used as statistical significant, evaluated by using one-way ANOVA followed by Student's *t*-test.

## 3. Result and Discussion

### 3.1. Blood Glucose Level

The fasting blood glucose levels in four-hour fasted rats were measured by directly using calibrated Glucometer (Dr. Morepen) with the help of strips. The glucose levels were recorded on days 01, 07, 14, and 21. The observed values given in Table 1 show that the test drug treatment group of animal reduces significant blood glucose level (89.01 ± 2.21 mg/dL) in comparison with standard treatment group (i.e., 130.06 ± 3.12 mg/dL) after oral administration of both freshly prepared test and standard drugs at the dose of 50 mg/kg body weight. It was almost comparable in the reduction of blood glucose level when compared with normal control at first day (85.06 ± 1.10).

### 3.2. Physical Parameters

The physical parameters like body temperature, body weight, and body behavior activities were performed in all treatment groups. The body temperature was comparable in all treatment groups when compared with normal and disease control group of rats. While the slight changes were observed in the body weight, the variation was nonsignificant in all the groups of rat. The body behavior activity was also observed as normal in all the treatment groups as well as controlled group of animals.

### 3.3. Percentage Wound Healing

**Figure 1:**
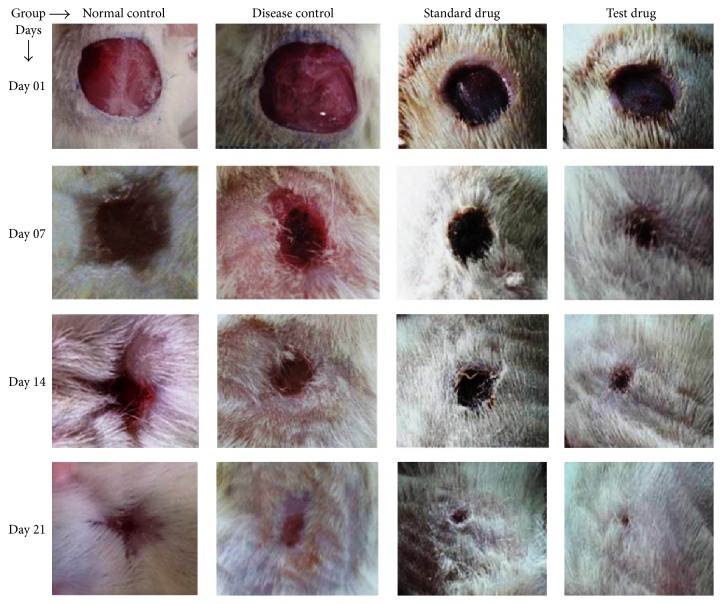
Percentage of wound healing in all experimental groups.

The opened area of the incised wound has been observed as a significant decrease on day 07 and even more on day 14 of all treatment groups (see Figure 1). Animals of Group III and Group IV showed an increased percentage of wound contraction at the end of the study when compared to diseased control group.* Chrozophora tinctoria* treated group of rats also showed an increase in the rate of wound reduction which leads to quick healing as established by decreased period of epithelialization (14.67 ± 0.28 days) when compared to disease control wounds that is 19.83 ± 0.37 days (Table 2).

Figure 1 shows that the synthesized collagen molecules were laid down by the side of the wound and converted into cross linkage to form fibers. Wound strength was acquired from both remodeling of collagen and the development of stable intra- and intermolecular cross linkage. Since granulation tissue from the incised space wounds treated with the hydroalcoholic leaves extract of* C. tinctoria* showed larger tensile strength, it does not only increase collagen synthesis per cell but also aids in cross-linking of the protein.

### 3.4. Hydroxyproline (Collagen) Content

This collagen content showed significant improvement in* C. tinctoria* leaves extract treatment group when compared with control group, while the standard treatment group did not show any significant improvements in the same condition (Table 3).

### 3.5. Total Protein Count

*C. tinctoria* leave extract-treated group showed considerable increase in total protein content when compared to control animals, while the standard treatment group showed slightest increase in total protein content in the same condition (Table 3).

### 3.6. Tensile Strength

In incised tissue model, the excisions of granulomas from subcutaneous implants were performed on the 14th day of wound formation. The breaking strength of the piece measuring about 15 mm in length and 8 mm in width was determined by a Constant Water Flow Technique for the given time period. The standard drug treated group (Group III) showed significant increase in tensile strength of granulation tissue of incised wounds when compared to the control group. On the other hand, Group IV showed more increase in tensile strength of granulation tissue of incised wounds in the same environmental condition (Table 3).

### 3.7. Tissue Granulation Weight

The tissue granulation weights were observed in two forms: one is the Wet Granulation Weight (WGW) and the second is Dry Granulation Weight (DGW). Table 3 showed that a significant increase in both Wet Granulation Weight and Dry Granulation Weight was observed in leaves extract-treated group when compared to control animals. Meanwhile the standard treatment group showed satisfactory increase in both tissue granulation weights in the same environment at the end of study [26].

### 3.8. Histopathological Analysis

Histopathological analysis of incised tissues of wound was collected with surgical removal process. The wound tissue was removed from the rat's body and then placed in a fixative to prevent the further interruption of tissues. The thickness of incision of wound skin tissue samples was fixed in 10% neutral buffered formalin (NBF) at 4°C for analysis [27].

**Figure 2:**
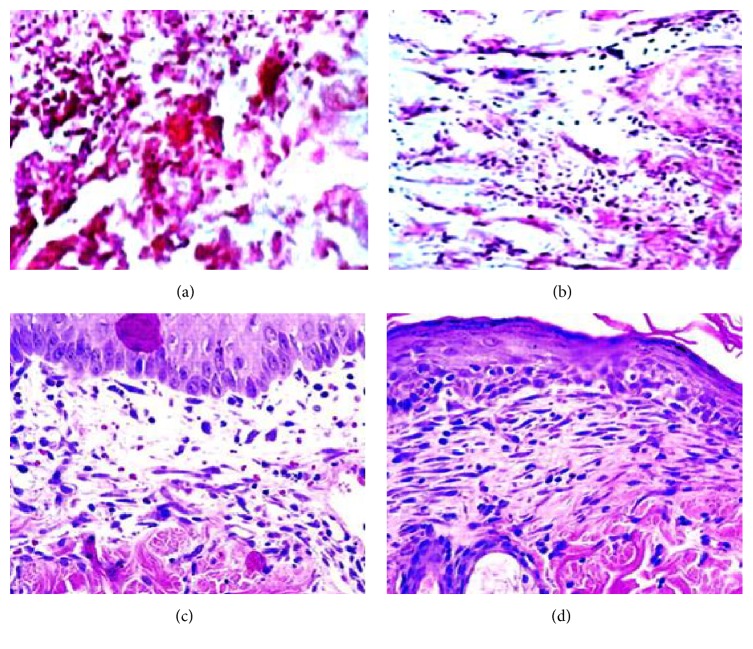
Quantitative histological evaluation of epithelial gap (EG), dermal gap (DG), and granulation tissue (GT) area was observed in one animal of all the treatment groups at the end of the study. The wounds in the test drug treated group showed significant improvement in closure compared to the other treatment groups. (a) Group I (normal control); (b) Group II (disease control); (c) Group III (standard control); and (d) Group IV (treatment control).

Histopathology of wounds (Figure 2) showed simple tissue separation, without evidence of tissue reaction and the absence of fibrin in the borders of the incision inflicted 30 min after cutting of tissue in striking contrast with the fibrin accumulation seen after 30 min of vital reaction. Despite their essential physiological role and obvious medical importance, we examined the growth and morphology during the wound healing process. In the peripheral part of the granulation tissue at the edge of the wound and at the depth, a transient appearance of lymphatic vessels was observed. The visualized lymphatic elements completely disappeared and were nondetectable at the end of the study, primarily due to the fact of diabetic wound.

## 4. Discussion

Wound examination is one of the most indispensable areas for the detection of pathogens. In the determination of wound, age plays a role in connection with traumatic deaths due to sharp and blunt force injuries. Elevated blood glucose level is well known to be connected with a variety of alterations in the connective tissue metabolism, in which the appearance of diabetes is the problem for poor wound healing. Loss of collagen observed in diabetes may be due to the decreased levels of production or improved catabolism of newly synthesized collagen or both.

As* Chrozophora tinctoria* was reported on the basis of wound healing effects, it was felt that it would be interesting to study the influence of the wound healing in diabetic conditions. Collagen is the major extracellular protein in the granulation tissue of wound healing and there is a quick increase in the synthesis of this protein in the wound area immediately after an injury. In addition it provides strength and reliability to a tissue matrix. Collagen also plays an important role in homeostasis and is required in subsequent epithelialization. Results obtained from the present study recommended that the treatment of wound in diabetic rats with hydroalcoholic leaves extract of* C. tinctoria* may be probably beneficial for the control of wound healing because it improves the maximum levels of collagen in the granulation tissues.

It is reported that an incision skin wound was healed by the growth of granulation tissue and reepithelialization. In the present work, the difference was observed at the time of wound healing, 2nd day after the skin incision in rats: reepithelialization of the wound surface was achieved, and early granulation tissue with angiogenic blood vessels appeared in the dermis. On the 7th day, neovascularization was maximal with the blood vessels appearing vertically, and collagen fibrils started to appear. Wound contraction was observed on the 14th day, while the blood vessels were less vertical. The blood vessels that regenerated in the granulation tissue of the rat skin incision wound were vertically running on the 7th day after the incision and changed into regular-shaped vessels by the 21st day.

It is well known that flavonoids have been used to antagonize lipid peroxidation that regularly occurs in case of physical injury/wound. Similarly antioxidants like vitamin C and vitamin E, several drugs that antagonize lipid peroxidation, help in increasing circulation and collagen viability. The phytochemical observation of* C. tinctoria* reported that the phytoconstituents containing flavonoids, terpenoids, steroids, tannins, glycosides, and so forth possess antimicrobial activity and also wound healing capability.

## 5. Conclusion

The hydroalcoholic leaves extract of* Chrozophora tinctoria *was used as wound healing activity in diabetic rat's model. This action is potentiated due to the presence of active chemical constituents that is chrozophorin, apigenin, rutin, and acacetin. It is a diverse array of active principles, which are able to target multiple mechanism involved in the pathophysiology of faster wound healing like fibroplasia, collagen synthesis, and contraction. Large numbers of cell types including neutrophils, macrophages, lymphocytes, keratinocytes, fibroblasts, and endothelial cells are also involved in this process.

Diabetes is one of the critical conditions, which opposes the faster wound healing process. The leaves extract of* C. tinctoria* has been observed to reduce the blood glucose level after oral administration of Streptozotocin. This effect is potentiated due to the presence of alkaloids and tannin since it has well-known coloring properties. Researchers already reported that flavonoids glycosides are well known for its anti-inflammatory activity as well as potentiating wound healing properties. Finally the present study accomplishes that the wound healing process can be enhanced by the use of hydroalcoholic leaves extract of* C. tinctoria* given through oral and dermal route of administration. It plays a significant protective role against physically damaged tissues in diabetic rats and fairly improved healing of wounds.

## Figures and Tables

**Table 1:** Effect of *C. tinctoria* on blood glucose levels after single dose administration of STZ.

Treatment	Blood glucose levels (mg/dL)			
	Day 01	Day 07	Day 14	Day 21
Group I	85.06 ± 1.10	83.08 ± 2.00	81.84 ± 1.80	80.06 ± 1.20
Group II	124.04 ± 3.02	117.32 ± 2.56	108.45 ± 3.00	92.78 ± 2.34
Group III	120.22 ± 2.34^*∗*^	125.14 ± 3.11^*∗*^	128.21 ± 2.88^*∗*^	130.06 ± 3.12^*∗*^
Group IV	125.46 ± 2.08^*∗*^	117.68 ± 2.45^*∗*^	107.87 ± 2.67^*∗*^	89.01 ± 2.21^*∗*^

Values are expressed as mean ± SEM (*n* = 6). Data were analyzed by using one-way ANOVA followed by Student's *t*-test. The values represent ^*∗*^ *P* < 0.05 considered as statistically significant.

**Table 2:** Effect of *C. tinctoria* on percentage wound healing of all experimental groups.

Treatment	% of wound healing				Epithelialization period (days)
	Day 01	Day 07	Day 14	Day 21	
Group I	10.02 ± 0.53	26.66 ± 0.91	54.87 ± 0.68	81.35 ± 0.42	20.37 ± 0.40
Group II	11.35 ± 0.53	32.17 ± 0.89	60.99 ± 0.36	87.71 ± 0.26	19.83 ± 0.37
Group III	06.54 ± 0.61^*∗*^	29.67 ± 0.27^*∗*^	68.27 ± 0.29^*∗*^	92.19 ± 0.24^*∗*^	16.05 ± 0.32^*∗*^
Group IV	14.54 ± 0.46^*∗*^	41.18 ± 3.59^*∗*^	79.72 ± 0.42^*∗*^	97.75 ± 0.35^*∗*^	14.67 ± 0.28^*∗*^

Values are expressed as mean ± SEM (*n* = 6). Data were analyzed by using one-way ANOVA followed by Student's *t*-test. The values represent ^*∗*^ *P* < 0.05 considered as statistically significant.

**Table 3:** Effect of hydroalcoholic leaves extract of *C. tinctoria* on collagen content, total protein count, tensile strength, and granulation weight (wet and dry) of incision wound.

Treatment	Collagen content (*μ*g)	Total protein count	Tensile strength	WGW (mg)	DGW (mg)
Group I	87.34 ± 2.17	105.42 ± 2.45	35.15 ± 2.40	77.49 ± 2.05	21.58 ± 1.88
Group II	94.10 ± 3.41	106.10 ± 3.29	36.85 ± 2.64	75.54 ± 2.65	24.85 ± 1.45
Group III	100.12 ± 2.09^*∗*^	115.09 ± 2.53^*∗*^	60.85 ± 2.84^*∗*^	90.63 ± 2.35^*∗*^	28.44 ± 1.45^*∗*^
Group IV	117.09 ± 2.39^*∗*^	128.12 ± 1.59^*∗*^	93.21 ± 3.24^*∗*^	122.8 ± 4.15^*∗*^	37.55 ± 1.62^*∗*^

Values are expressed as mean ± SEM (*n* = 6). Data were analyzed by using one-way ANOVA followed by Student's *t*-test. The values represent ^*∗*^ *P* < 0.05 considered as statistically significant.
